# Twenty-Seventh Annual Meeting American Medical Association

**Published:** 1876-07

**Authors:** 


Twenty-Seventh Annual Meeting of the American Medical Association.—The Association met at Horticultural Hall, Philadelphia, Tuesday, June 7th, and was called to order at eleven o'clock A.M. by Dr. Wm. Pepper, chairman of the Committee of Arrangements. A prayer was then offered by the Rev. Dr. Beadle, after which Dr. W. K. Bowling, of Kentucky, introduced the President elect, Dr. J. Marion Sims, of New York. The latter was supported by the following officers of the Association: Vice-Presidents, Drs. Sam. Lilly, of New Jersey; Ninian Pinckney, U. S. N.; and S. D. Seelye, of Alabama; Dr. W. B. Atkinson, Secretary; and Dr. R. W. Dunglison, Assistant Secretary.

Dr. Pepper, on behalf of the Committee of Arrangements, delivered an address of welcome to the delegates, in which he referred to the attractive medical features of Philadelphia, its sanitary resources, its medical schools and organizations, concluding with a resolution inviting the following gentlemen to seats upon the platform: Surgeon-general Wm. Roth, Twelfth Corps (Royal Saxon) German Army, and staff, viz: Assistant-surgeon Hans Heyman, Twelfth Corps (Royal Saxon) German Army, and Dr. Max Brille, Dresden, Germany. Members by invitation: Dr. Wywoodzoff, of St. Petersburg, of the Russian Commission; Le Docteur Gaffrey, representatives of the Paris Press to the Exposition; Surgeon-general J. K. Barnes, United States Army.

The reading of the roll of registered delegates was dispensed with after some discussion, when the secretary read a letter from Dr. William O. Baldwin, late President of the Association, regretting his inability to be present.

*Miscellaneous Business.*—After a vote of thanks had been tendered for the address, Dr. Pepper then announced that the medical building, directly behind the judges' hall in the Landsdowne Ravine, would be used as a place of rendezvous for physicians visiting the centennial.

The times and places of meeting of the sections were next announced.

Dr. Toner, of Washington, moved that ten o'clock on Wednesday be set apart for the reading of the Address on Surgery.

Dr. Toner called upon the Secretary to read the list of the reports to be presented, which was done. A motion was then made to adjourn until three o'clock, and, at the same time an effort was made to change the place of meeting to some other hall, but without success. The meeting then adjourned to nine o'clock on Wednesday.

*Evening Entertainment.*—During the evening the members accepted the invitation of the Committee of Arrangements to a grand promenade concert and supper at Horticultural Hall. The attendance was large and the occasion enjoyable to all.

SECOND DAY.—*Committee on Nominations.*—The meeting was called to order by the President pursuant to previous adjournment.

The following Committee on Nominations was appointed:

Alabama, L. B. Sulze; Arkansas, George T. Hood; California, W. Baker; Connecticut, A. Woodward; Delaware, W. Marshall; District of Columbia, J. Elliott; Florida, G. W. Betton; Georgia, J. P. Logan; Indiana, L. Humphrey; Illinois, T. D. Washburne; Iowa, W. Watson; Kentucky, H. M. Skillman; Kansas, C. V. Mattson; Michigan, A. S. Heaton; Massachusetts, A. B. Hall; Minnesota, E. C. Cross; Maryland, Thos, F. Latimer; Maine, A. B. Snow; Missouri, John T. Hodgen; Mississippi, Wm. Campton; New Hampshire, J. L. Swett; New York, N. C. Husted; New Jersey, S. Lilly; North Carolina, E. Grissom; Ohio, W. J. Scott; Pennsyvania, Traill Green; Rhode Island, L. C. Butler; South Carolina, Jones McIntosh; Tennessee, John H. Callender; Texas, E. Darnell; Vermont, W. D. Holton; Virginia, J. S. Wellford; West Virginia, J. C. Hupp; Wisconsin, H. P. Strong; United States Army, J. R. Smith; United States Navy, A. L. Gihon.

The Judicial Council reported that they had decided that the delegates from the Arkansas State Medical Society were entitled to recognition.

*Natural Purifiers—Water-Supply and Sewerage.*—Dr. R. C. Kedzie, of Lansing, Mich., read a paper, with the above title. He contended that the conditions of water and air were the main if not the only ones upon which the discussion of his subject rested, but the purifier *par excellence* was oxygen.

In conclusion the gentleman offered the following resolutions, which were adopted.

*Resolved,* That it is the first duty of states and municipalities—first in importance, and first in the order of time—to make a sanitary survey of the water supply to preserve it against all unnecessary and avoidable contaminations.

*Resolved*, That no municipality should introduce a water system without at the same time providing a corresponding and extensive sewer system.

Doctors Wilhelm Hiorth and H. G. Hoist, Medical Directors, Christiania, Sweden, were made members by invitation.

*Centennial Surgery*.—Dr. A. Garcelon, of Maine, read an address on the Progress of Surgery during the past century. * * * Drs. Seguin and Bowditch were appointed additional delegates to the International Congress.

*Reports of Treasurer, Publication and Library Committees.*—The report of the Treasurer showed that there was an unexpended balance of $4,577 07 in his hand to date.

The report of the Committee of Publication stated that of the volume of the Transactions for 1875 nine hundred and fifty copies were printed, at an aggregate cost of $2,000. Of these 833 copies have been distributed to members, 28 to medical journals and societies, leaving 39 on hand.

The report of the Librarian stated that during the past year there had been added to the library 124 distinct titles, exclusive of yearly volumes of transactions of societies, reports of hospitals and boards of health, and volumes of medical journals, where these have been previously catalogued as distinct titles. This addition makes the library consist at present of 630 distinct titles, which comprehend 1,514 volumes, including pamphlets.

The report of Dr. C. C. Howard, Delegate to the Brussels International Medical Congress, held in September, was presented, and referred to the Committee on Publication.

A sketch, prepared by Prof. Hailes, of the life of Dr. Armsby, of Albany, N. Y., on motion, referred to the Committee on Publication.

The meeting then adjourned until 9:30 o'clock A.M., on Thursday.

The afternoon was occupied in the meeting of the different sections.

THIRD DAY.—At 9:30 A.M. Dr. Sims called the meeting to order pursuant to previous adjournment.

On motion, the resolution of the day before, that all persons whose names are at present on the roll shall be voted members, was reconsidered, and it was agreed, after some discussion, that the roll of members, amounting to 730 names, be called. In accordance with this desire the Secretary began calling the roll, and continued to do so until ten o'clock, when the hour arrived for the reading of the Address on Obstetrics, by Dr. S. Busey, of Washington, D. C.

On the conclusion of the reading Dr. J. L. Atlee moved that the address be referred to the Committee on Publication, for publication, and discussed at the next meeting of the session on Obstetrics. Adopted.

The effort was then again made to do away with the reading of the roll, but unsuccessfully. The motion occasioned considerable discussion.

*Michigan State Society and Delegateship.*—On the reading of the roll, at the name of a delegate from the State Society of Michigan, the delegate was objected to, as coming from a society against which charges were pending. At this juncture the report of the Judicial Council, signed by Dr. Benham, Chairman, recommending that the delegates from Michigan State Society be received, was presented. The report was applauded and the reading of the roll was continued.

*Members in Absentio.*—At the name of Dr. Thomas J. Griffiths, of Louisville, an objection was made that he was not or had been present, and the Secretary was requested to strike his name from the roll if the objection should be found, on inquiry, to be well founded.

*Female Representation.*—On reading the name of Sarah Hackett Stevens, representing the Illinois State Society, Dr. Brodie, of Detroit, moved that that and all such names be referred to the Judicial Council. A motion that this resolution be laid upon the table was carried by a large vote, amid considerable applause.

The President asked if the vote was intended to recognize her right to a seat, when loud cries of "Yes," and cheers, emphatically answered the question.

Dr. Toner, of Washington, moved that the roll as read be confirmed, with the exception of the objection.

*The Question of Patents in Medicine.*—A resolution providing that it was not derogatory in any physician to take out a patent for a surgical instrument of any kind, was referred to the Judicial Council.

*The McDowell Memorial Fund.*—Dr. Keller, of Kentucky, on behalf of the Trustees of the fund for a monument to the late Dr. McDowell, of Kentucky, reported a recommendation for an increase of one dollar each year in membership dues, the amount thus raised to go to said fund.

Dr. Toner stated that the resolution so recommending would have to lay over one year, under the rules. He hoped, however, that the rules would be waived for this purpose by unanimous consent.

Objection was raised and a motion to lay the resolution on the table was agreed to.

Dr. Toner moved that $1,000 be appropriated out of the funds of the Association for the McDowell fund.

Dr. Howard moved the point of order that it was the rule of the Association that no new business should be transacted except on the first and fourth days of the session of the Association, and hoped the rules would not be departed from.

The chair decided the order well taken, and the motion of Dr. Toner was declared out of order.

Dr. Sims said he was as anxious as any one to secure the money asked for, but after conversation with gentlemen around him he was satisfied the subject could not be discussed to-day. The Association would have to defer action until to-morrow.

Dr. Henry A. Martin, of Boston, moved that a committee be appointed by the chairman to take into consideration the subject of bovine vaccination as compared with the usual arm to arm practice, to report at the next annual meeting. The motion was adopted, after which the Association adjourned till 9:30 A.M. on Friday.

The sections held their usual meeting in the afternoon, and completed the business before them.

The evening was spent on the part of the members in responding to invitations to the receptions of the following Philadelphia physicians and surgeons; Drs. D. Hayes, Agnew, J. Solis Cohen, Louis A. Duhring, H. Lenox Hodge, John H. Packard, William H. Pancoast, William Pepper, and Ellwood Wilson.

Fourth Day—The closing session of the Association was held in Horticultural Hall, and was called to order by the President at 9:30 a.m.

A charge against the State Society of Illinois was referred to the Judicial Council.

*The Graduation of "Irregular" Persons*—Dr. Toner, of Washington, offered the following, which was unanimously adopted:

*Resolved*, That members of the medical profession who in any way aid or abet the graduation of medical students in irregular or exclusive system of medicine, are deemed thereby to violate the spirit of the ethics of the American Medical Association.

*State Boards of Health in Existence*—Dr. W. B. Atkinson, the secretary, presented a report which stated that in obedience to the resolution adopted at the session of 1875, he reports that in reply to his inquiries he is informed that boards of health exist in Alabama, California, Georgia, Massachusetts, Michigan, Minnesota, Virginia, and Wisconsin. He had writton to the Governors of Delaware, Indiana, Iowa, Nebraska, New Jersey, New York, South Carolina, Texas, and Vermont, with almost negative results.

*Catalogue of National Library*—Dr. H. C. Wood, of Philadelphia, offered the following, which was adopted:

*Resolved*, That a committee of three be appointed by the chair to obtain from Congress an appropriation for the publication of the Subject Catalogue of the National Library, and that the State societies are requested to take such action as may be deemed fit to further said object.

*Officers Elected for* 1877—The Nominating Committee next presented the following report through their chairman, which was duly adopted:

President—Dr. Henry J. Bowditch, of Massachusetts.

Vice Presidents—Dr. N. J. Pittman, of North Carolina; Dr. Franklin Staples, of Minnesota; Dr. Joseph R. Smith, of U. S. Army; Dr. Samuel C. Busey, of Washington, D. C.

Treasurer—Dr. Casper Wister, of Pennsylvania.

Librarian—Dr. William Lee, of District Columbia.

Committee on Library—Dr. Jchnson Eliot, of District of Columbia.

Assistant Secretary—Dr. J. H. Hollister, of Illinois.

Committee of Arrangements—Drs. N S. Davis, S. W. Freer, H. A. Johnson, T. D. Fitch, H. W. Jones, Joseph P. Ross, Leslie Curtis.

Committee of Publication—Dr. W. B. Atkinson, chairman; Dr. T. M. Drysdall, Albert Fricke, Samuel D. Gross, Casper Wister, Richard J. Dunglison, all of Pennsylvania; and Dr. Williams, of District of Columbia.

Next place of meeting, Chicago, Ill. Time of meeting, first Tuesday in June, 1877.

Judicial Council—Drs. N. S. Davis, of Illinois; E. L. Howard, of Maryland; W. O. Baldwin, of Alabama; H. W. Dean, of New York; A. N. Talley, of South Carolina; J. P. Logan, of Georgia; and D. W. Stormont, of Kansas, in place of the seven whose terms expire at this meeting. The rest of the present council continue.

Committee on Prize Essays—Dr. N. S. Davis, Illinois, chairman; Edmund Andrews, E. Ingalls, Moses Gunn, E. P. Cook, all of Illinois.

Special Committee on Influence of Climate in Pulmonary Diseases in Florida—Dr. E. T. Sabal, continued.

*Delegates to International Medical Congress*—Delegates to the International Medical Congress to be held September 4, 1876, at Philadelphia: Drs. H. I. Bowditch, Massachusetts; E. Seguin, New York; Thomas L. Maddox, Iowa; J. S. Welford, Virginia; A. Dunlap, Ohio; John T. Hedgen, Missouri; Joseph Carson, Pennsylvania; John C. Dalton, New York; W. O. Baldwin, Alabama; D. W. Yandell, Kentucky; N. S. Davis, Illinois; Austin Flint, Sr., New York, T. G. Richardson, Louisiana; W. F. Westmoreland, Ga.; A. M. Pollock, Pennsylvania; Frank Hastings Hamilton, New York; G. M. Bemiss, Louisiana; L. A. Dugas, Georgia; Francis Bacon, Connecticut; Hunter McGuire, Virginia; A. G. Shortleff, California; E. M. Moore, New York; O. W. Holmes, Massachusetts; G. A. Otis, U. S. Army; F. E. Gunnell, U. S. Navy; E. C. Harwood, New York.

*Salary of the Permanent Secretary*—On motion of Dr. J. L. Atlee, of Lancaster, the salary of Dr. W. B. Atkinson, the permanent Secretary, was made $1,000 for the past year.

*The Necessity for State Boards of Health*—Dr. Bell, of Iowa, offered the following, which was adopted:

*Resolved*, That there be appointed a committee of three persons, members of this Association, in each of those States where there has been no action taken for the establishment of boards of health, to urge upon those States the necessity of the establishment of such boards.

*The United States Pharmacopœa*—Dr. E. R. Squibb, of Brooklyn, N. Y., remarked upon the practicability of revising the Pharmacopœia, after which he offered the following, which were adopted:

WHEREAS, The usual time for a decennial revision of the United States Pharmacopœia is drawing near; and

WHEREAS, The plan of revision and publication in force since 1820 may not now be the best that could be devised; therefore, be it

*Resolved*, That the American Medical Association take the whole subject of the National Pharmacopœia into consideration for a revision of its management, and for the present time, with especial reference to the following question:

First—Whether the present plan of decennial revision and publication be practically sufficient for the needs of the materia medica and pharmacy of the present time; and if not sufficient, whether a plan could be devised which might offer probable advantages enough to justify an attempt to disturb the present one?

Second—Whether this Association be the proper custodian in this country of the interests involved in the National Pharmacopœia; and if it is the proper source of the National Codex, whom can it invite to co-operate with it in the work?

Third—If it be a work for this Association, in what way can its details be wisely undertaken, with any prospect of material improvement upon the present plan?

*Resolved*, That in order to facilitate mature and general deliberations upon so important a subject, the final discussion of these resolutions be laid over at least one year; and that the matter be recommended to the President of the Association for consideration in his annual address for the meeting of 1877.

On motion, the thanks of the Association were returned to the Messrs. Kiralfy, proprietors of the Alhambra Theatre, for the loan of their beautiful building on Thursday.

The Association then adjourned *sine die*.—*New York Medical Record.*

